# The Structuring of Sage (*Salvia officinalis* L.) Extract-Incorporating Edible Zein-Based Materials with Antioxidant and Antibacterial Functionality by Solvent Casting versus Electrospinning

**DOI:** 10.3390/foods11030390

**Published:** 2022-01-29

**Authors:** Ana Salević, Dušica Stojanović, Steva Lević, Milena Pantić, Verica Đorđević, Radojica Pešić, Branko Bugarski, Vladimir Pavlović, Petar Uskoković, Viktor Nedović

**Affiliations:** 1 Department of Food Technology and Biochemistry, Faculty of Agriculture, University of Belgrade, Nemanjina 6, 11080 Belgrade, Serbia; ana.salevic@agrif.bg.ac.rs (A.S.); slevic@agrif.bg.ac.rs (S.L.); milenas@agrif.bg.ac.rs (M.P.); vlaver@agrif.bg.ac.rs (V.P.); 2 Department of Materials Science and Engineering, Faculty of Technology and Metallurgy, University of Belgrade, Karnegijeva 4, 11000 Belgrade, Serbia; duca@tmf.bg.ac.rs (D.S.); puskokovic@tmf.bg.ac.rs (P.U.); 3 Department of Chemical Engineering, Faculty of Technology and Metallurgy, University of Belgrade, Karnegijeva 4, 11000 Belgrade, Serbia; vmanojlovic@tmf.bg.ac.rs (V.Đ.); rpesic@tmf.bg.ac.rs (R.P.); branko@tmf.bg.ac.rs (B.B.)

**Keywords:** zein, sage extract, electrospinning, solvent casting, incorporation, fiber mats, films, antioxidant activity, antimicrobial activity, active edible packaging

## Abstract

In this study, in order to develop zein-based, edible, functional food-contact materials in different forms incorporating sage extract (10, 20, and 30%), solvent casting and electrospinning were employed. The study aimed to assess the effects of the applied techniques and the extract’s incorporation on the materials’ properties. The solvent casting generated continuous and compact films, where the extract’s incorporation provided more homogenous surfaces. The electrospinning resulted in non-woven mats composed of ribbon-like fibers in the range of 1.275–1.829 µm, while the extract’s incorporation provided thinner and branched fibers. The results indicated the compatibility between the materials’ constituents, and efficient and homogenous extract incorporation within the zein matrices, with more probable interactions occurring during the solvent casting. All of the formulations had a high dry matter content, whereas the mats and the formulations incorporating the extract had higher solubility and swelling in water. The films and mats presented similar DPPH^•^ and ABTS^•+^ radical scavenging abilities, while the influence on *Staphylococcus aureus* and *Salmonella enterica* subsp. *enterica* serovar Typhimurium bacteria, and the growth inhibition, were complex. The antioxidant and antibacterial activity of the materials were more potent after the extract’s incorporation. Overall, the results highlight the potential of the developed edible materials for use as food-contact materials with active/bioactive functionality.

## 1. Introduction

Modern lifestyles, market globalization, new products, and demands concerning food quality, safety, consumers’ health and environmental issues have triggered innovations in the food packaging sector [1]. As is known, the leading causes of food deterioration are oxidation and microbial spoilage. These processes can decrease the nutritional value of food, negatively affect its organoleptic properties, and increase the risk of food-borne illnesses. The challenges of overcoming these problems; improving the quality, safety, functionality, and shelf-life of food; minimizing food losses; and reducing the total environmental footprint gave rise to novel food packaging concepts [2].

The emerging concept intended to extend shelf life, and maintain or even improve the condition of packaged food is active packaging, which is designed to deliberately incorporate components that would release or absorb substances into or from the packaged food or its surrounding environment [3]. This packaging concept thus implies the extension of the traditional, passive role in the protection and marketing of a product to an innovative, active role in food quality and safety improvement [4,5]. In addition, a novel trend is the development of edible, functional food packaging materials which are designed to enhance the health-beneficial properties of food. The packaging materials within this approach act as matrices to incorporate bioactive or functional compounds, and to retain active properties before their release into food [6,7]. The incorporation of bioactive or functional compounds within a packaging structure represents a promising approach for their use, as these compounds are sensitive, prone to degradation, may be incompatible with the food matrix, or affect food’s organoleptic properties. Thus, this approach combines the principles of encapsulation and packaging technologies aiming to protect bioactive or functional compounds, overcome their drawbacks for implementation in food products, increase their performance, and facilitate the development of new products [7,8].

Because the usual thermal processing of polymers can cause the degradation of bioactive or functional compounds, there is a need to consider alternative techniques for the design and development of food-contact materials, or surfaces, incorporating these compounds [9]. A widely used technique to produce active, edible films at a laboratory scale is solvent casting, as it is simple and does not require specific equipment. It involves the direct incorporation of active compounds into a film-forming solution, then spreading the solution onto a level plate, followed by drying under controlled conditions to remove the solvent, resulting in film formation. New approaches are developing this technique to scale-up the manufacturing of films with larger dimensions, and to decrease the time and energy consumption [10,11,12]. Another technique of high interest for the incorporation of active compounds within the packaging polymer matrix and the development of functional materials for added-value food is electrospinning. This technique involves electrohydrodynamic processing based on the action of a high-voltage electric field imposed on a solution containing polymer and active compounds. It induces charge and shear stress on the surface of the solution droplet at a capillary tip. The forces established in the electric field cause the ejection of the charged solution jet from the capillary tip towards the grounded collector, jet stretching, and rapid solvent evaporation, resulting in solid fibers at the micron-, submicron-, or nanoscale deposited on the collector as non-woven mats. The interest in this technique relies on the process properties (continuous, facile, cost-effective, flexible, non-mechanical, nonthermal), as well as the structural and functional properties of the fibers (a high surface to volume ratio, tailored morphology and size). In addition, novel design solutions for the equipment and process allow mass fiber production [13,14,15].

Solvent casting and electrospinning are both versatile regarding the biopolymers which are suitable for processing [11,15]. The use of biodegradable polymers from natural, renewable sources is of great interest due to ever-growing environmental concerns related to the traditionally used petroleum-derived polymeric packaging materials, primarily regarding the depletion of natural resources and waste-accumulation problems. In this respect, biopolymer-based packaging materials represent a potential sustainable replacement for conventional polymeric materials for specific applications [12,16,17]. The revaluation of agro-industrial by-products as resources for the development of bio-based materials is of interest from economic and environmental perspectives [16]. Zein was chosen in this study to develop edible, functional materials, as it is a biodegradable and biocompatible polymer derived from renewable resources. It is a corn prolamin protein and a by-product of corn-processing industries. The amino acid residues of zein are predominantly hydrophobic, but there are also hydrophilic ones, which allow its compatibility with many other polymers and various active compounds. In addition, it has good film-forming, electrospinnability, gas and moisture-barrier properties, and the ability to act as a carrier for hydrophobic and hydrophilic active compounds. These properties, taken together, highlight the potential of zein as a promising biopolymer for the design of edible, active and bioactive films and coating materials [18,19,20].

The nature of active compounds for incorporation within packaging materials is diverse, but the focus is on the natural ones over synthetic ones [21]. This approach supports the trend of the replacement of chemical additives and the development of “green” products [22]. In this sense, phytochemicals extracted from herbs and spices known for their preservative and health-beneficial properties, have gained great interest [1]. One of these herbs is sage (*Salvia officinalis* L.), which is used in food preparation due to its flavoring and seasoning properties, but also in medicine due to its pharmacological activities [23]. As has been reported, sage extracts can induce antimicrobial effects against various bacteria and fungi, and antioxidant effects against different free radicals and oxidative hemolysis [22,24,25]. In addition, sage extracts have been shown not to be hepatotoxic [24]. Due to these preservative and bioactive properties, the sage extract was chosen as a model active constituent for incorporation within the biopolymeric matrix in our study. For example, Akcan et al. [26] showed that the sage extract’s incorporation within cast films based on whey protein isolate contributed to the films’ effectiveness in the antioxidant protection of cooked meatballs during frozen storage. According to the best of our knowledge, there is no study on a zein matrix incorporating the sage extract, regardless of the technique used for the material’s synthesis.

Given the above, this study aimed to develop active and bioactive zein-based materials incorporating the sage extract through a green synthesis approach for potential application as edible, functional food-contact materials. Solvent casting and electrospinning were employed to structure different zein matrices incorporating the sage extract. The incorporation of different sage extract contents by both techniques was also studied. The study also aimed to assess and compare the effects of the applied techniques, the sage extract’s incorporation, and its content on the properties of the resulting materials. In this context, the materials were analyzed and compared regarding their morphology, matrix–extract interactions, water-resistance (dry matter, solubility, swelling), and functional (antioxidant and antibacterial) properties.

## 2. Materials and Methods

### 2.1. Materials and Reagents

All of the chemicals were of analytical or HPLC grade, and were used as received, without any purification. Plant material (sage, *Salvia officinalis* L., *Salviae folium*) was acquired from the Institute for Medicinal Plant Research “Dr. Josif Pančić” (Belgrade, Serbia). The biopolymer, i.e., zein, was supplied by Acros Organics (Geel, Belgium). The solvents used were ethanol (Reahem, Novi Sad, Serbia), acetic acid (Zorka Pharma-hemija, Šabac, Serbia), and hydrochloric acid (Sigma Aldrich, St. Louis, MO, USA). The salts were potassium bromide (Fisher Scientific, Waltham, MA, USA); potassium persulphate, disodium hydrogen phosphate, and sodium chloride (Sigma Aldrich, St. Louis, MO, USA); and sodium acetate and sodium dihydrogen phosphate monohydrate (Merck KGaA, Darmstadt, Germany). The reagents for the antioxidant assays were 2,2,-diphenyl-1-picrylhydrazyl, 2,2′-azinobis(3-ethylbenzothiazoline-6-sulphonic acid), and 6-hydroxy-2,5,7,8-tetramethyl chromane-2-carboxylic acid (Sigma Aldrich, St. Louis, MO, USA). The microbiological media used were Müller Hinton Agar and Müller Hinton Broth (Merck KGaA, Darmstadt, Germany).

### 2.2. Synthesis of the Zein-Based Materials

#### 2.2.1. Preparation of the Sage Extract

The maceration technique using an aqueous ethanol solution (50% *v/v*) as the extraction medium was employed to prepare the sage extract. The ratio of the plant material to the solvent was 1:20 (*w*/*v*). The extraction was carried out on an orbital shaker (model 3005, GFL, Burgwedel, Germany) by continuous shaking fixed at 200 rpm at room temperature for 90 minutes. After the extraction, the solid residues were removed from the extract solution using a tea strainer. Finally, the solvent was evaporated from the filtrate at 35 °C in a universal oven (model UF55, Memmert GmbH + Co.KG, Schwabach, Germany) to obtain the dry extract for the active material synthesis.

#### 2.2.2. Preparation of the Solutions

Four solution formulations based on zein were prepared: a plain solution (25% *w*/*w* zein, without the sage extract loading, control), and three solutions loaded with different contents of the sage extract (10, 20, and 30% *w*/*w,* based on the zein content). For the plain solution preparation, zein was dissolved in an aqueous ethanol solution (80% *v*/*v*) under constant magnetic stirring (2 h) at room temperature. Regarding the active solutions’ preparation, the required extract amount was dissolved in 80% *v/v* aqueous ethanol first by ultrasonic waves (40 kHz) for 15 min, and then by magnetic stirring for 24 h at room temperature. Subsequently, zein was added into the solutions whilst maintaining the constant magnetic stirring (2 h) to ensure complete dissolution. The prepared solutions were processed using solvent casting and electrospinning techniques to fabricate the materials.

#### 2.2.3. Solvent Casting

The prepared zein-based solutions (10 g), plain and loaded with the different sage extract contents, were spread onto leveled polystyrene Petri dishes (9 cm in diameter) and dried at 35 °C in the universal oven for 48 h. The resulting solvent-cast films—plain and those incorporating 10, 20, and 30% (*w*/*w*) extract—were designated as Zs.c., Z-E10s.c., Z-E20s.c., and Z-E30s.c., respectively.

#### 2.2.4. Electrospinning

The solutions—the same as were used above for the solvent casting—were processed by the single-needle, vertical electrospinning setup CH-01 (Linari Engineering, Pisa, Italy). Plastic syringes (20 mL) loaded with the zein-based solutions were mounted onto the syringe pump and connected to a metallic needle (with a 0.8-mm inner diameter) via a polytetrafluoroethylene (PTFE) tube. The positive electrode of the high-voltage power supply was attached to the needle. Preliminary screening studies were carried out regarding the processing parameters in order to obtain cone-jet stability and the production of bead-free fibers. All of the solutions were electrospun at a constant flow rate of 2 mL/h onto a grounded metallic collector covered with an aluminum foil sheet fixed at 13 cm from the needle tip. The applied voltage for the solutions containing 0, 10, 20, and 30% sage extract were 18, 20, 22, and 22 kV, respectively. The electrospinning process occurred in a closed chamber at room conditions for 1 h for each sample. The fabricated electrospun mats—plain and incorporating 10, 20, and 30% (*w*/*w*) extract—were designated as Ze., Z-E10e., Z-E20e., and Z-E30e., respectively.

### 2.3. Characterization of the Solvent-Cast Films and the Electrospun Mats

#### 2.3.1. Morphology

The morphological properties of the solvent-cast films and the electrospun mats were analyzed by scanning electron microscopy (SEM), employing a JEOL JSM-6390LV microscope (JEOL Ltd., Tokyo, Japan). The samples were first sputtered with gold under vacuum in a BAL-TEC SCD 005 sputter coater (BAL-TEC AG, Balzers, Liechtenstein), and thereafter were observed at an accelerating voltage of 15 kV. The size of the electrospun fibers was measured from the SEM micrographs using the Image J program (National Institutes of Health, Bethesda, MD, USA). The measurement of 100 randomly selected fibers from 4 micrographs was performed for each formulation in order to determine the size distribution and the average size.

#### 2.3.2. Fourier Transform Infrared (FT-IR) Spectroscopy

The material constituents in their native forms and the developed materials were analyzed by FT-IR spectroscopy using an IRAffinity-1 spectrometer (Shimadzu, Kyoto, Japan). Before the analysis, the samples were homogenously mixed with potassium bromide, compressed into pellets, and then used in that form. The FT-IR spectra were collected in the wavenumber range of 4000–500 cm^−1^, at a resolution of 4 cm^−1^, from 100 scan accumulations.

#### 2.3.3. Dry Matter Content, Water Solubility, and Swelling Degree

The materials’ dry matter content, water solubility, and swelling degree were gravimetrically determined. The solvent-cast film and the electrospun mat specimens of 2 × 2 cm in triplicate were weighted with a precision of ±0.0001 g to obtain the initial sample mass (*m*_1_). After being dried in a conventional oven at 105 °C to reach a constant mass (initial dry mass, *m*_2_), the samples were immersed in 50 mL distilled water, covered, and agitated at 100 rpm at room temperature for 24 h on the orbital shaker. Afterward, the samples were taken out from the water, weighed (*m*_3_), and dried to a constant mass (final dry mass, *m*_4_). The materials’ dry matter content, water solubility, and swelling degree were calculated according to Equations (1)–(3), respectively.

(1) $$\mathrm{Dry}\;\mathrm{matter}\;\mathrm{content}\;\left(\%\right)=\frac{m_{2}}{m_{1}}\times 100,$$

(2) $$\mathrm{Solubility}\;=\frac{\left(m_{2}-m_{4}\right)}{m_{2}}\times 100,$$

(3) $$\mathrm{Swelling}\;\mathrm{degree}\;=\frac{\left(m_{3}-m_{2}\right)}{m_{2}}\times 100.$$

#### 2.3.4. Antioxidant Activity

The free radical scavenging ability of the solvent-cast films and the electrospun mats was determined in order to evaluate their antioxidant activity. In order to this end, spectrophotometric assays with 2,2,-diphenyl-1-picrylhydrazyl (DPPH^•^) radicals and 2,2′-Azino-bis(3-ethylbenzothiazoline-6-sulfonic acid) (ABTS^•+^) radical cations were employed. Before the analysis, the materials (25 mg) were dissolved in 3 ml ethanol aqueous solution (80% *v*/*v*).

The DPPH^•^ radical scavenging assay was performed according to the previously described methodology [27,28], with some modifications. In order to prepare the DPPH^•^ working solution, DPPH^•^ in ethanol (1.86 × 10^−4^ mol/L) and acetic buffer (0.1 mol/L, pH = 4.3) were mixed at a volume ratio of 2:1. An aliquot of the DPPH^•^ working solution (1.9 mL) was added to 0.1 mL of the previously dissolved and appropriately diluted samples. The blank was prepared by adding 1.9 mL of the DPPH^•^ working solution to 0.1 mL of the solvent. After the reaction mixtures were stored in the dark at room temperature for 30 minutes, the absorbance was measured at 525 nm using a UV/Vis spectrophotometer (model HALO DB-20S, Dynamica Scientific Ltd., Livingston, UK). The DPPH^•^ free radical inhibition was calculated according to the following equation:

(4) $$I\;\left(\%\right)=\frac{A_{b}-A_{s}}{A_{b}}\times 100,$$

where *A_b_* and *A_s_* are the absorbance values of the blank and the sample, respectively.

6-hydroxy-2,5,7,8-tetramethyl chromane-2-carboxylic acid (Trolox, 0–1 mol/L) was used as a standard to create the calibration curve *I* (%) = *f* (Trolox concentration). Employing this calibration curve, the antioxidant activity of the samples was determined and expressed as µmol Trolox equivalents (TE)/g of the sample. The assay was performed in triplicate.

The ABTS^•+^ radical cation decolorization assay was conducted according to the methodology described by Re et al. [29], with some modifications. In order to prepare the ABTS^•+^ solution, stock ABTS solution (14 mmol/L) and potassium persulphate (4.9 mmol/L)—both in phosphate buffer (5 mmol/L, pH = 7.4)—were mixed at a volume ratio of 1:1, and stored in the dark at room temperature for 16 h. Before the analysis, the ABTS^•+^ solution was diluted with the phosphate buffer to reach an absorbance of 0.70 ± 0.02 at 734 nm. An aliquot of this working ABTS^•+^ solution (3 mL) was added to 30 µL of the previously dissolved and appropriately diluted samples. The blank contained 30 µL of the solvent instead of the sample. After the reaction mixtures were stored in the dark at room temperature for 6 minutes, the absorbance was measured at 734 nm. The inhibition of the ABTS^•+^ radical cation was calculated using Equation (4). Calibration curve *I* (%) = *f* (Trolox concentration) was prepared with Trolox (0–2.5 mmol/L) as a standard, and was used to determine the antioxidant activity of the samples. The results were expressed as µmol Trolox equivalents (TE)/g of the sample. The measurements were performed in triplicate.

#### 2.3.5. Antibacterial Activity

A modified broth macrodilution test was applied to evaluate the antibacterial potential of the developed materials [30,31]. The tested bacterial strains were *Staphylococcus aureus* (ATCC 25923) and *Salmonella enterica* subsp. *enterica* serovar Typhimurium (ATCC 14028), which were obtained from the American Type Culture Collection (ATCC, Manassas, VA, USA). For the cultures’ preparation, the bacterial colonies from the Müller Hinton Agar (MHA) were suspended in Müller Hinton Broth and incubated overnight at 37 °C. After the incubation, the bacterial suspensions were diluted to 10^5^–10^6^ CFU/mL and then used for the antibacterial activity test. Before the experiment, the materials were sterilized by exposure on each side to ultraviolet light for 15 min in a biosafety cabinet (model BSC-1800IIA2-X, BIOBASE, Jinan, China). For the antibacterial activity testing, the samples of the materials were placed in sterile cups and inoculated with the bacterial suspensions at a concentration of 200 mg/mL. Tubes containing only the bacterial suspensions were prepared as the controls. After incubation at 37 °C for 24 h, serial dilutions were prepared, plated onto MHA, and again incubated at 37 °C for 24 h. Finally, the colony-forming units (CFU) corresponding to viable bacterial counts were enumerated. The antibacterial activity of the materials (R) was determined as a reduction of log_10_(CFU/mL) of the tested bacteria during the incubation, according to Equation (5):

(5) $$R={\;\log}_{10}\left(\frac{Nc}{Nt}\right),$$

where *Nc* and *Nt* represent the average number of viable bacterial cells after 24 h of incubation with the control and the tested materials, respectively.

### 2.4. Statistical Analysis

The morphological (fiber size), water resistance (dry matter, solubility, swelling), and functional (antioxidant and antibacterial) properties of the developed materials were statistically analyzed using SPSS Statistics 26 software package (IBM, Endicott, NY, USA). The results were presented as the mean value ± the standard deviation. The data were subjected to a one-way analysis of variance (ANOVA) to evaluate the effects of the applied techniques and the sage extract incorporation, separately, on the materials’ properties. A post-hoc Tukey’s HSD test at a 95% confidence level (*p* < 0.05) was employed to identify the statistically significant differences between the materials.

## 3. Results and Discussion

In the present study, solvent casting and electrospinning techniques allowed the successful structuring of zein-based materials in two different forms. Different formulations—a plain formulation (control, without extract) and formulations incorporating the sage extract at different loadings (10, 20, and 30%, *w*/*w*)—were prepared in order to understand the effects of the extract’s incorporation and the applied techniques on the development of materials with antioxidant and antibacterial functionality. The electrospinning process was mainly stable and continuous, with no dripping of the solutions or formation of beads or fibers with sizes surpassing the micron range. Apart from the fabrication of the electrospun mats, it was possible to cast films from the same solutions. The effects of the sage extract’s incorporation within the zein matrix—depending on the applied techniques—on the structural, physicochemical, and functional properties of the resulting materials were analyzed.

### 3.1. Morphological Properties

**Figure 1:**
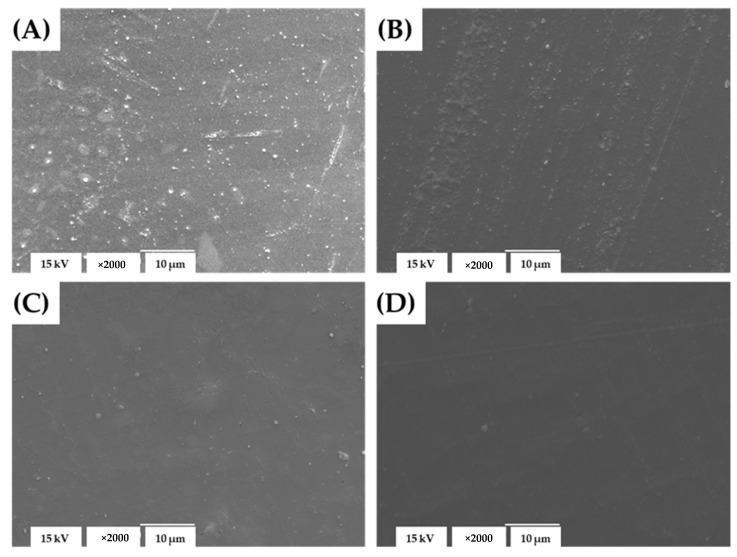
The SEM micrographs of the zein-based solvent-cast materials: plain film (**A**) and films incorporating 10 (**B**), 20 (**C**), and 30% (**D**) of the sage extract.

The microstructure of the solvent-cast and electrospun zein-based materials—plain and loaded with the sage extract—was examined by SEM. The surface morphology of the materials obtained by the solvent casting technique is shown in Figure 1. All of the formulations of the cast materials presented structures of continuous and compact films. However, some differences in the surface morphology of the films were observed depending on the composition. Among the films obtained here, the plain film had a rough surface. The sage extract’s incorporation changed the surface morphology, and resulted in a smoother and more homogenous film surface, which was more evident as the content of the incorporated extract increased. As was reported, the films’ microstructures were affected by the intramolecular aggregation of polymeric chains, the spatial organization, and the interactions of the constituents during the synthesis [32,33]. In this regard, the smoother surface of the zein films incorporating the sage extract compared to the plain zein film indicates possible interactions between the matrix and the extract constituents that yield more regular and homogeneous depositions of the proteins’ chains during the drying. A similar effect of the production of smoother and more compact protein film structures was reported for cast gelatin films after green tea extract’s incorporation, which was related to protein–polyphenol interactions [34]. In addition, no phase separation, pores, or cracks were observed in any of the SEM micrographs of the cast films, regardless of the extract’s incorporation and its contents. These microstructure properties point to the compatibility between the films’ constituents, efficient incorporation, and the uniform dispersion of the sage extract within the zein matrix.

**Figure 2:**
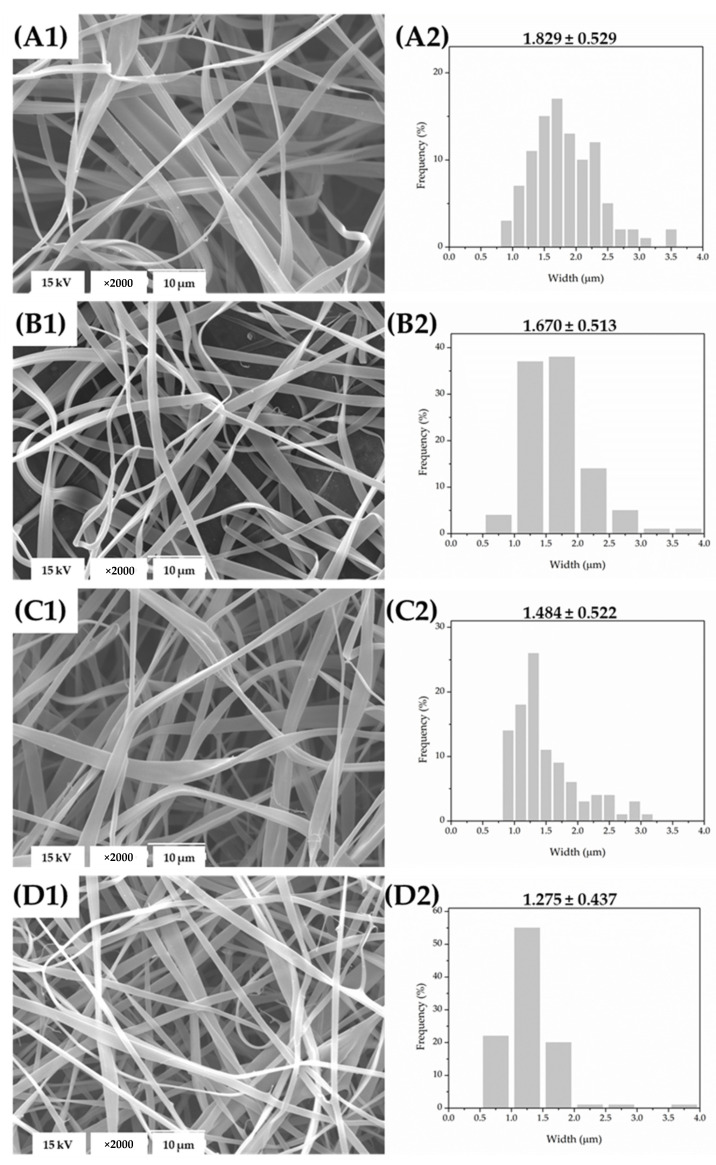
The SEM micrographs of the zein-based electrospun materials (**A1**,**B1**,**C1**,**D1**) and the width distribution histograms (**A2**,**B2**,**C2**,**D2**): plain mat (**A**) and mats incorporating 10 (**B**), 20 (**C**), and 30% (**D**) of the sage extract.

The SEM micrographs of the materials produced by the electrospinning technique are presented in Figure 2(A1,B1,C1,D1). As one can observe, there was a distinct difference in morphology between the solvent-cast and electrospun materials. The prepared solutions and the applied electrospinning processing parameters enabled chain entanglement in the electric field, resulting in non-woven fibrous mats. Thus, the electrospun mats of all of the formulations were structurally composed of dense, randomly oriented fibers with smooth surfaces and ribbon-like structures, with no beads or aggregates. The ribbon-like structure of zein fibers electrospun from aqueous ethanol solution was also observed by Neo et al. [35], and was ascribed to high solvent volatility. More precisely, the rapid solvent evaporation during the electrospinning process forms a thin skin that collapses during the remaining solvent evaporation, resulting in the ribbon-like fibers [36]. In general, the morphological properties of the here-obtained electrospun mats suggest the efficient and homogenous dispersion of the extract within the zein fibrous matrix. There was an influence of the sage extract’s incorporation on the morphology of the resulting fibers. Thus, the periodical instability of the electrospinning process for the solution containing 30% of the extract resulted in branched fibers (Figure 2(D1)). This indicates that the balance between the surface tension and electrical forces is altered by this extract content, resulting in jet instability [36]. Because of these effects, higher extract loadings (>30%) were not tested. In general, the morphology of the electrospun mats was influenced less by their composition when compared to the solvent-cast films due to the remarkably different kinetics of these techniques [37].

The effect of the sage extract’s incorporation on the width distribution and average width size of the resulting fibers is shown in the histograms gathered in Figure 2(A2,B2,C2,D2). The extract’s incorporation and the increase in the loaded content induced decreased the average width size of the fibers. It is well known that the processing parameters, solution properties, and ambient conditions influence the electrospinning process, and the fibers’ morphology and size [38,39]. As previously stated in Section 2.2.4, the applied voltage was increased in order to fabricate the electrospun mats from the solutions containing the extract compared to the plain (control) zein solution, while other processing parameters were held constant. The voltage was increased to improve the spinnability of these solutions and the stability of the process. A higher voltage may increase the strength of the electric field and the electrostatic repulsive forces, thereby causing a higher degree of jet stretching during the electrospinning process [39,40]. Therefore, the decreased width of the fibers incorporating the sage extract may be due to the higher voltage which was applied. Similarly, carvacrol’s incorporation resulted in a decrease of the average zein fiber’s diameter, but it also resulted in the appearance of beads [41]. Altan et al. also showed that the average diameter of the zein fibers incorporating carvacrol decreased as the carvacrol content increased [42]. They attributed this reduction in the fiber diameter—caused by the incorporation of the active compound—to decreased solution viscosity and thus to the greater stretching forces of the jet.

### 3.2. Fourier Transform Infrared (FT-IR) Spectroscopy

**Figure 3:**
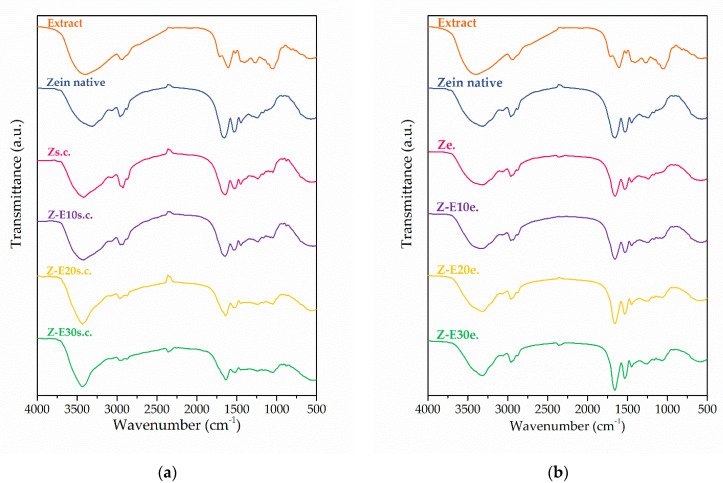
FT-IR spectra of the unloaded sage extract, native zein, and zein-based solvent-cast (**a**) and electrospun (**b**) materials.

The FT-IR spectroscopy analysis was employed to assess the chemical composition, the potential interactions between the biopolymeric matrix and the incorporated extract, and the structure at the molecular level. Figure 3 gathers the FT-IR spectra recorded for the materials’ constituents in their native forms and the resulting electrospun and solvent-cast materials.

The unincorporated extract in its dry form exhibited several characteristic bands in the spectrum. The broadband in the range of 3600–3000 cm^−1^ and the band at 2925 cm^−1^ correspond to the stretching vibrations of phenolic O–H and aliphatic C–H groups, respectively [43]. The bands at 1715 cm^−1^ (C=O stretching vibrations) and 1057 cm^−1^ (C–O and C–C stretching vibrations) most probably originate from the extracted plant primary metabolites [44]. The presence of secondary plant metabolites, primarily phenolic compounds, may be observed in the bands at 1609 cm^−1^, 1519 cm^−1^, 1408 cm^−1^ (aromatic ring stretching and bending vibrations), and 1267 cm^−1^ (C-H and O-H bending vibrations) [45,46].

The spectrum of the matrix in its native form, before processing, showed characteristic bands for zein at 3319 cm^−1^ (N-H and O–H stretching vibrations, amide A), 1657 cm^−1^ (C=O stretching vibrations, amide I), and 1533 cm^−1^ (N-H bending and C–N stretching vibrations, amide II). Other bands were observed at 2961 cm^−1^ (C–H stretching vibrations), 1449 cm^−1^ (NH_3_^+^ symmetric deformations), and 1239 cm^−1^ (C–N axial deformation vibrations, amide III) [18,33,45]. The spectra of the solvent-cast film and the electrospun mat without the incorporated extract also exhibited these characteristic bands for zein. However, the applied techniques influenced the zein spectrum. In the case of the plain film, there were some spectral changes in the bands’ positions compared to the native zein. Namely, the positions of the bands related to amide A and amide I were shifted to 3418 and 1645 cm^−1^, respectively. On the other hand, there were no significant variations in the bands’ positions on the plain mat and the native zein spectra. These observations indicate the existence of different molecular structures in the solvent-cast and the electrospun materials in addition to the previously discussed morphological differences. The reason for this may be the much slower rate of solvent evaporation during the materials’ fabrication by solvent casting, rather than by electrospinning. Thus, intramolecular interactions between the biopolymer chains are more probable during solvent casting.

In the case of the solvent-cast films, the sage extract’s incorporation provoked changes in the bands’ positions compared to those in the plain film. In particular, the increase in the incorporated content promoted shifts of the bands related to amide A, amide I, and amid II, and a slight narrowing of amide A, indicating interactions between the extract compounds and the biopolymeric matrix. Thus, in the case of the film incorporating 30% extract, the band positions of amide A, amide I, and amide II were shifted to 3432, 1638, and 1524 cm^−1^, respectively. As reported in the literature, these spectral variations demonstrate intermolecular interactions through hydrogen bonding between zein and phenolic compounds of the extract and changes in the protein structure [42,46]. These interactions might be responsible for the previously observed, more-compact morphology of the film surfaces incorporating the extract. Concerning the electrospun materials, the extract’s incorporation did not induce significant variations in the band positions of amide A, amide I, and amide II compared to the plain mat, suggesting the absence of strong interactions between the extract compounds and the matrix in this case. The only variations were observed for the band at 1238 cm^−1^ (1260 cm^−1^ for the mat incorporating 30% extract) as an indicator of slight interactions. Similarly to the above assumption, the solvent rate evaporation might influence the interactions between the biopolymeric matrix and the extract compounds. Thus, the rapid solvent evaporation during electrospinning may not allow enough time for the establishment of interactions between the materials’ constituents, in contrast to solvent casting. Overall, after the sage extract’s incorporation within the zein matrix, no new bands appeared on any of the spectra of the resulting materials, except the weak molecular interactions which were already mentioned. The spectra of these materials showed only already mentioned bands related to the matrix overlapping the bands associated with the extract compounds, regardless of the applied technique or the incorporated extract content. These observations indicate the efficient incorporation of the sage extract within the zein matrix. In addition, there was no band splitting, suggesting the compatibility and uniform dispersion of the materials’ constituents.

### 3.3. Dry Matter Content, Water Solubility, and Swelling Degree

The physical properties of the zein-based materials—regarding their dry matter content, solubility, and a swelling degree in water as a function of the sage extract’s incorporation and applied techniques—were assayed. Table 1 shows the obtained results.

Dry matter content is an important property, given that it indirectly reflects the moisture content of the materials and gives an insight into their stability under environmental humidity and ability to maintain the moisture of food [47]. In general, all of the developed formulations of the materials presented a high dry matter content. As is evident, the dry matter content of the zein materials was not significantly influenced (*p* > 0.05) by the extract’s incorporation or the applied techniques. Thus, the dry matter content was primarily related to the zein and its hydrophobic nature. In addition, the obtained results suggest that the organization of the zein chains allowed easy solvent evaporation during the drying process.

The water solubility and swelling degree are other important properties of materials that provide an insight into their behavior in an aqueous environment, and into their water resistance [48,49]. From an application viewpoint as edible and biodegradable packaging materials, the water solubility also determines the rate of the materials’ breakdown during consumption and biodegradation [47,50]. All of the formulations of the developed films and mats swelled, but maintained their integrity after being immersed in water under constant agitation for 24 h. The plain film and mat, without incorporated extract, had the lowest solubility and swelling degree in water due to the high content of nonpolar amino acid residues in the zein [51]. As can be observed, the extract’s incorporation within the zein matrix significantly increased (*p* < 0.05) the water solubility and swelling degree of the resulting materials. This behavior could be related to hydrophilic groups of the extract’s constituents that might have interacted with the water molecules, thus increasing the affinity of the materials toward the water and promoting the release of the extract into the aqueous medium [49,52]. Mushtaq et al. [53] also experienced an increase in the water solubility of zein films (from ~6 to ~18%) after their incorporation with pomegranate peel extract. Likewise, a higher swelling degree in water (from ~9 to ~52%) was found for zein film incorporated with monolaurin and *Zataria multiflora* Boiss. essential oil as active compounds [54]. Compared to the films based on some of the frequently used biopolymers, the materials developed in our study were less soluble and more swollen than chitosan film incorporating rosemary extract (28% solubility and a 170% swelling degree) [55] and starch film incorporating purple sweet potato anthocyanin (60% solubility and a 148% swelling degree) [56].

Among the formulations of the materials developed here, the electrospun mats were more prone to solubility and swelling in water when compared to the solvent-cast films. The difference in the water affinity between the films and the mats might be due to the previously discussed structural differences resulting from the applied technique. In this regard, the porous structure of the electrospun mats might allow higher water intrusion, diffusion, and uptake than the compact structure of the solvent-cast films. In general, the obtained results highlighted the potential of zein for use as a matrix for the development of water-absorbing materials which are not readily soluble.

### 3.4. Antioxidant Activity

DPPH^•^ radical and ABTS^•+^ radical cation scavenging ability assays were employed in order to determine the antioxidant activity of the solvent-cast films and the electrospun mats. The employed radicals were nitrogen-centered radical species that are widely used as indicators to test the hydrogen-donating capacity—and thus the antioxidant activity—of phytochemicals [57]. Table 2 gathers the DPPH^•^ and ABTS^•+^ scavenging ability of the zein-based materials. The obtained materials showed an ability to scavenge both radicals in a composition-dependent manner. In this context, the zein film and mat without incorporated extract exhibited the lowest antioxidant activity. The inherent antioxidant activity of zein may originate from some of its amino acids residues and short peptides acting as free radical scavengers, and from xanthophyll pigments with characteristic chemical structures making them antioxidants [58]. The sage extract’s incorporation enhanced (*p* < 0.05) the ability of the zein-based materials to scavenge DPPH^•^ and ABTS^•+^ radicals. In particular, the increase in the extract-loaded content resulted in materials with a more potent (*p* < 0.05) radical scavenging ability, regardless of the applied technique or radical species. This positive contribution of the sage extract’s incorporation to the ability of the resulting materials to scavenge the free radicals is in agreement with the reported antioxidant activity of the unloaded extract [25,59], which is correlated to phenolic compounds [59,60]. The antioxidative properties of phenolic compounds are related to their chemical structures. In this regard, aromatic rings can stabilize and delocalize unpaired electrons, while hydroxyl groups along the aromatic ring have the potential to donate hydrogen or electrons to free radicals [61]. Previous studies also reported an increase in the antioxidant activity of the protein films due to the incorporation of natural extracts which are rich in phenolic compounds [34,62].

As may be noted, the samples exhibited different efficiencies of scavenging ability for DPPH^•^ and ABTS^•+^ radical species. These differences can be ascribed to the different solubility and accessibility of the radical species that influence hydrogen or electron transfer from antioxidants and radical stabilization by antioxidants [63].

As shown in Table 2, the materials’ DPPH^•^ and ABTS^•+^ radical scavenging ability were solely affected by their composition, i.e., the extract’s incorporation and the loaded content. The films and mats with the same composition showed similar (*p* > 0.05) antioxidant activity. Therefore, the influence of the applied techniques on the antioxidant activity of the resulting materials was negligible (*p* > 0.05). In this way, these results show that the antioxidant activity of the sage extract endured both solvent casting (48 h exposure to 35 °C) and electrospinning (exposure to high-voltage) processing. As the total antioxidant activity was determined, implying the dissolution of the materials before the testing, the materials’ structure had no influence. In general, the obtained results highlighted that the sage extract primarily contributed to the ability of the materials to scavenge the radical species due to its inherent antioxidant activity, which was retained during the materials’ fabrication. Therefore, the sage extract’s incorporation within the zein matrix may be an effective strategy to boost the activity and functional properties of the resulting materials in the suppression of reactive radical species and the prevention of oxidation reactions.

### 3.5. Antibacterial Activity

In this section, the antibacterial activity of the developed zein-based materials was evaluated regarding their potential to reduce viable bacterial counts when exposed to direct contact at 37 °C for 24 h. *S. aureus* and *S. enterica* serovar Typhimurium, which are foodborne pathogens, were tested as target bacteria. Table 3 shows the results of the antibacterial activity test for the zein materials as a function of the sage extract’s incorporation and the applied techniques.

All of the developed formulations of the materials, regardless of the extract’s incorporation or the applied technique, exhibited an ability to reduce the growth of *S. aureus*. The plain zein solvent-cast film and electrospun mat, without incorporated extract, showed the lowest antibacterial activity, as expected. As was previously reported, the zein mat [64] and film [65] without any active compounds did not affect *S. aureus* growth. Given this, the slightly expressed antibacterial activity of the here-developed plain materials seemed not to be the activity of zein itself, but rather a result of the high swelling degree that might restrict the bacterium growth. The sage extract’s incorporation and the increase in its content enhanced (*p* < 0.05) the antibacterial activity of the resulting materials. This effect is due to the previously shown inherent antibacterial activity of the extract [25]. The antibacterial activities of medicinal and dietary plant extracts, including sage, are in a highly linear relationship with their total phenolic content [66]. The exact mechanism of the antibacterial activity of these compounds remains undefined. These compounds are presumed to cause irreversible damage to the cell wall; change the structure, function, and permeability of the cytoplasmic membrane; interact with intracellular components; inhibit the intracellular enzymes; and reduce the intracellular ATP content and suppress its synthesis [67,68]. Similarly to our study, other authors have also reported an increase in the antibacterial efficiency of protein-based materials with an increase in the content of various incorporated active compounds against *S. aureus*. Some of the examples are a zein mat loaded with curcumin (20–40%) [69], soy protein isolate film incorporating cortex *Phellodendron* extract (10–22.5%) [70], and gelatin film containing *Zataria multiflora* essential oil (2–8%) [71].

As can be further observed, the materials with the same composition showed different antibacterial activity depending on the applied technique. In particular, the electrospun mats were more potent against the growth of *S. aureus* compared to the solvent-cast films with the same composition. Of note, the electrospun mats and the solvent-cast films retained their integrity after being incubated with the bacterial culture. In this regard, the electrospun mats and the solvent-cast films have different surface antibacterial efficiencies, which could be due to the previously discussed structural differences. As was reported, the porous fibrous mats have a much lower density and a higher surface-area-to-volume ratio when compared to the solid films of the same size [72]. In addition to this, the electrospun mats have a higher swelling degree than the solvent-cast films, as discussed in Section 3.3. Thus, the higher surface-area-to-volume ratio and swelling degree of the mats could provide more contact and interactions with the bacteria, resulting in better antibacterial performance when compared to the films.

In general, materials are considered antimicrobial (i.e., bactericidal) for a value of the reduction in colony-forming units of a minimum of 3 log_10_(CFU/mL) [73,74]. In this regard, the results presented in Table 3 suggest a bacteriostatic effect on *S. aureus* provided by the material formulations Z.s.c., Z-E10s.c., Z-E20s.c., Ze., and Z-E10e. On the other hand, bactericidal effect (R > 3) against *S. aureus* was determined when exposed to the material formulations Z-E30s.c., Z-E20e., and Z-E30e.

The plain zein-based electrospun mat and solvent-cast film influenced the growth not only of *S. aureus* but also *S*. *enterica* serovar Typhimurium (Table 3). As we already discussed in the case of *S. aureus*, the antibacterial activity of the materials without incorporated extract could be due to their swelling, which reduces cells’ growth by restricting—i.e., absorbing—the available water and nutrients. Similarly to the results for antibacterial activity against *S. aureus*, the extract’s incorporation and increase in the loaded content induced a more potent activity to the mats and the films against *S*. *enterica* serovar Typhimurium due to the intrinsic antibacterial activity of sage. In contrast to *S. aureus*, *S*. *enterica* serovar Typhimurium was more susceptible to the solvent-cast films, and less susceptible to the electrospun mats. In particular, all of the solvent-cast film formulations exhibited a bactericidal (R > 3) effect against *S*. *enterica* serovar Typhimurium. The electrospun mat formulations exhibited bacteriostatic effects against *S*. *enterica* serovar Typhimurium. The different susceptibility of *S. aureus* and *S*. *enterica* serovar Typhimurium towards films and mats implies a complex antibacterial action which is influenced by the structural properties of both materials and bacteria. As is known, numerous factors may influence the antibacterial activity of the materials. Thus, the surface and chemical properties of the films and the mats may cause variations in the antimicrobial activity. According to Tarus et al. [75], the chemical properties and morphology (the density and shape of fibers) of cast films and fibrous mats influence the convenient locations for the growth and colonization of cells, and the interference determining their antimicrobial activity. In addition, the adhesion and colonization of bacteria on a fibrous substrate—and consequently the antibacterial activity of the fibrous mats—are affected by the size of the fibers and the size and shape of the tested bacteria [76]. These findings highlight the complexity of the antibacterial action of the materials, and explain the various susceptibilities of the tested bacteria towards the different materials.

The discussed results, taken together, revealed the potential of the sage extract’s incorporation within the zein matrix to impart both antibacterial and antioxidant functionality to the resulting materials. These results point out the potential of the developed edible materials for use as active/bioactive food-contact materials. However, further research is needed to clearly define the interference of the different materials with different bacteria, as well as the mode of their antibacterial action.

## 4. Conclusions

In this study, solvent casting and electrospinning techniques allowed the successful structuring of edible zein-based materials in two different forms incorporating different contents of the sage extract through a green synthesis approach. The analysis of the materials gave an insight into the effects of the applied techniques and the plant extract’s incorporation on their properties. The solvent casting and electrospinning yielded distinctly different materials. The cast materials structurally presented continuous and compact films with a more homogenous surface morphology as the content of the incorporated extract increased. On the other hand, the electrospun materials structurally presented non-woven mats composed of dense, randomly oriented, ribbon-like fibers. The extract’s incorporation and the increase in its content resulted in thinner fibers, and provoked their branching. The materials’ characterization suggested the compatibility between the constituents, efficient incorporation, and the uniform dispersion of the sage extract within the zein matrices. More probable intermolecular and intramolecular interactions occurred during solvent casting than during electrospinning. The materials had a high dry matter content regardless of the extract’s incorporation or the applied techniques. In addition, the materials showed water-absorbing potential without ready solubility, and the formulations incorporating the extract compared to the plain ones, and the electrospun mats compared to the cast films, were more prone to swelling and solubility in water. The extract’s incorporation and the increase in its content induced a more potent ability of the materials to scavenge DPPH^•^ and ABTS^•+^ radicals, and to inhibit the growth of *S. aureus* and *S*. *enterica* serovar Typhimurium bacteria. The efficiency of the sage extract’s incorporation within the zein matrices in boosting the activity and functional properties of the edible materials regarding the prevention of oxidation reactions and bacterial growth makes these materials interesting for use as active/bioactive food-contact materials.

## Figures and Tables

**Table 1:** Physical properties of the solvent-cast films and the electrospun mats.

Sample	Dry Matter Content (%)	Water Solubility (%)	Swelling Degree (%)
Zs.c.	92.05 ± 4.13 ^a^	8.42 ± 0.21 ^a^	194.38 ± 9.52 ^a^
Z-E10s.c.	92.12 ± 2.26 ^a^	12.51 ± 0.51 ^b^	180.64 ± 8.11 ^a^
Z-E20s.c.	90.02 ± 4.41 ^a^	13.89 ± 0.62 ^b,c^	293.99 ± 7.20 ^b^
Z-E30s.c.	90.07 ± 3.68 ^a^	12.57 ± 0.62 ^b^	326.35 ± 13.32 ^b^
Ze.	91.12 ± 3.72 ^a^	9.86 ± 0.24 ^a^	302.85 ± 7.42 ^b^
Z-E10e.	91.33 ± 2.24 ^a^	15.50 ± 0.63 ^c^	321.29 ± 15.74 ^b^
Z-E20e.	91.29 ± 4.47 ^a^	20.17 ± 0.99 ^d^	462.26 ± 20.76 ^c^
Z-E30e.	91.82 ± 4.12 ^a^	24.54 ± 1.10 ^e^	462.76 ± 18.89 ^c^

Different letters within the same columns indicate a significant difference (*p* < 0.05) among the samples.

**Table 2:** Antioxidant activity of the solvent-cast films and the electrospun mats.

Sample	DPPH^•^ Scavenging Ability (µmol TE/g)	ABTS^•+^ Scavenging Ability (µmol TE/g)
Zs.c.	45.55 ± 1.20 ^a^	102.14 ± 4.18 ^a^
Z-E10s.c.	178.82 ± 0.80 ^b^	283.30 ± 13.61 ^b^
Z-E20s.c.	290.88 ± 6.07 ^c^	363.55 ± 17.97 ^c^
Z-E30s.c.	373.93 ± 6.31 ^d^	454.07 ± 25.61 ^d^
Ze.	45.00 ± 1.20 ^a^	91.24 ± 3.44 ^a^
Z-E10e.	176.06 ± 3.06 ^b^	284.31 ± 16.12 ^b^
Z-E20e.	300.54 ± 6.24 ^c^	372.80 ± 20.41 ^c^
Z-E30e.	367.42 ± 9.69 ^d^	469.43 ± 34.72 ^d^

Different letters within the same columns indicate a significant difference (*p* < 0.05) among the samples.

**Table 3:** Antibacterial activity of the solvent-cast films and the electrospun mats against *S. aureus* and *S*. *enterica* serovar Typhimurium.

Sample	*S. aureus*		*S. enterica* Serovar Typhimurium	
	Bacterial Count log_10_(CFU/mL)	CFU Reduction R	Bacterial Count log_10_(CFU/mL)	CFU ReductionR
Control	10.45 ± 0.13 ^a^	-	10.51 ± 1.07 ^a^	-
Zs.c.	9.26 ± 0.02 ^b^	1.19	6.87 ± 0.07 ^b,c^	3.64
Z-E10s.c.	7.98 ± 0.16 ^c^	2.47	6.74 ± 0.20 ^b,c^	3.77
Z-E20s.c.	7.77 ± 0.18 ^c,d^	2.68	6.00 ± 0.00 ^b^	4.51
Z-E30s.c.	7.29 ± 0.05 ^e,f^	3.16	6.00 ± 0.00 ^b^	4.51
Ze.	9.21 ± 0.18 ^b^	1.24	10.35 ± 0.11 ^a^	0.16
Z-E10e.	7.52 ± 0.04 ^d,e^	2.93	9.74 ± 0.35 ^a,d^	0.77
Z-E20e.	7.42 ± 0.05 ^d,e,f^	3.03	8.47 ± 0.33 ^d,e^	2.04
Z-E30e.	7.01 ± 0.09 ^f^	3.44	8.13 ± 0.25 ^c,e^	2.38

Different letters within the same columns indicate a significant difference (*p* < 0.05) among the samples.

## Data Availability

Data is contained within the article.
